# In-vitro antiplatelet effect of melatonin in healthy individuals and patients with type 2 diabetes mellitus

**DOI:** 10.1007/s40618-023-02102-7

**Published:** 2023-05-06

**Authors:** A. Böhm, V. Lauko, K. Dostalova, I. Balanova, I. Varga, B. Bezak, N. Jajcay, R. Moravcik, L. Lazurova, P. Slezak, V. Mojto, M. Kollarova, K. Petrikova, K. Danova, M. Zeman

**Affiliations:** 1 Premedix Academy, Medená 18, 81102 Bratislava, Slovakia; 2 3rd Department of Internal Medicine, Faculty of Medicine, Comenius University in Bratislava, University Hospital Bratislava, Bratislava, Slovakia; 3 National Institute of Cardiovascular Diseases, Bratislava, Slovakia; 4 Slovak Medical University, Bratislava, Slovakia; 5 Cardio-Integra s.r.o., Bratislava, Slovakia; 6 Faculty of Medicine, Comenius University in Bratislava, Bratislava, Slovakia; 7 Department of Complex Systems, Institute of Computer Science, Czech Academy of Sciences, Prague 8, Czech Republic; 8 Department of Animal Physiology and Ethology, Faculty of Natural Sciences, Comenius University in Bratislava, Bratislava, Slovakia

**Keywords:** Melatonin, Acute myocardial infarction, Circadian variation, Diabetes mellitus, Platelet aggregation

## Abstract

**Purpose:**

The incidence of acute myocardial infarctions (AMI) shows circadian variation typically peaking during morning hours with a decline at night. However, this variation does not occur in patients with diabetes mellitus (DM). The night’s decline of AMI may be partially explained by melatonin-related platelet inhibition. Whether this effect is absent in diabetic patients is unknown. The aim was to study the effect of melatonin on in-vitro platelet aggregation in healthy individuals and patients with type 2 DM.

**Methods:**

Platelet aggregation was measured in blood samples from healthy individuals (*n* = 15) and type 2 DM patients (*n* = 15) using multiple electrode aggregometry. Adenosine diphosphate (ADP), arachidonic acid (ASPI) and thrombin (TRAP) were used as agonists. Aggregability for each subject was tested after adding melatonin in two concentrations.

**Results:**

In healthy individuals, melatonin inhibited platelet aggregation in both higher (10–5 M) and lower concentrations (10–9 M) induced by ADP, ASPI, and TRAP (*p* < 0.001, *p* = 0.002, *p* = 0.029, respectively). In DM patients, melatonin did not affect platelet aggregation in both concentrations induced by ADP, ASPI, and TRAP. Melatonin decreased platelet aggregation induced by ADP, ASPI, and TRAP significantly more in healthy individuals compared to patients with DM. (*p* = 0.005, *p* = 0.045 and *p* = 0.048, respectively).

**Conclusion:**

Platelet aggregation was inhibited by melatonin in healthy individuals. *In-vitro* antiplatelet effect of melatonin in type 2 DM patients is significantly attenuated.

## Introduction

Acute myocardial infarction (AMI) is a leading cause of mortality and morbidity in the developed world. Depending upon the infarction size, 30-day mortality is up to 6.5% [1–3]. From the surviving patients, 10% will die within 12 months and almost half of the patients will require rehospitalization within one year [4, 5].

The incidence of AMI shows a circadian variation that peaks during morning hours continuously declines in the afternoon, and reaches a trough during the evening and night-time. The increased morning incidence of AMI is most likely caused by a rise in blood pressure, heart rate, vascular tone and prothrombotic activity [6–9]. Interestingly, in the population of patients with diabetes mellitus (DM), circadian variation of AMI is absent [10]. We hypothesized this could be caused by the inability of melatonin to inhibit platelets aggregation in DM.

Melatonin is an endogenous hormone released primarily by the pineal gland and is one of the key components of the human circadian system [11, 12]. Melatonin directly or indirectly affects many physiological functions including the immune system, body temperature, foetal development, metabolism, coagulation [13–17] and platelet aggregation [18–20]. Evidence shows that genetic variants in the melatonin receptor as a result of single nucleotide polymorphisms are associated with atherosclerosis and the risk of myocardial infarction (MI) [21–23]. The relationship between melatonin and DM is also the subject of extensive research. Melatonin supplementation has been shown to improve insulin resistance, leptin resistance, hyperinsulinaemia, hyperglycaemia and reduce HbA1c levels. Low levels of melatonin secretion were able to predict the onset of Type 2 DM in women [24] and melatonin has been also studied as a potential drug in the therapeutic management of diabetic patients [25].

It is not known which platelet aggregation pathways are impaired by melatonin and if this effect is attenuated in DM. We designed a study to evaluate the effect of melatonin on platelet aggregation activated by arachidonic acid, adenosine diphosphate and thrombin in the blood of healthy individuals and in patients with type 2 DM.

## Methods

The study was conducted in accordance with the Declaration of Helsinki and approved by the Ethics Committee of the National Cardiovascular Institute, Bratislava, Slovakia. Written informed consent was obtained from all participants.

### Study population

Fifteen consecutive healthy adult individuals scheduled for blood donation were enrolled in the control normoglycemic group and 15 consecutive adult outpatients with type 2 DM on insulin were enrolled in the DM group. Exclusion criteria for both groups were treated with any antiplatelet, anticoagulant, or anti-inflammatory drug, smoking, present cancer, acute or chronic infectious disease, renal disease, pregnancy, history of any thrombotic cardiovascular disease, history of any platelet disorder or bleeding disorder and platelet count < 120 × 10^9^/L.

### Laboratory methods

In all participants, peripheral, fasting blood was taken from an antecubital vein in the morning after 30 min rest in a seating position. Blood was collected during the daytime when melatonin concentrations are very low (below 1 pg mL^−1^) [26]. These levels are negligible compared to night-time levels, which we mimicked in our study.

Blood for platelet counts was collected in 3.0 mL tubes containing K2EDTA and assessed by an automated haematology analyser SYSMEX XT 4000i.

Blood for platelet aggregation analysis was collected in 3.0 mL tubes containing hirudin and stored at room temperature for a minimum of 30 min and a maximum of two hours before analysis. Subsequently, blood samples were aliquoted and incubated with saline or melatonin in two different concentrations (10–5 and 10–9 M, respectively) for 10 min.

Platelet aggregation analysis was performed by multiple electrode aggregometry using the impedance-based Multiplate^®^ Analyzer (Roche, Mannheim, Germany). Arachidonic acid 15 mmol/L (ASPI), Adenosine diphosphate 0.2 mmol/L (ADP) and thrombin-receptor-activating-peptide (TRAP-6) 1 mmol/L (TRAP) were used as agonists (ASPItest, ADPtest and TRAPtest, Roche, Mannheim, Germany). Platelet aggregation levels are expressed as area under the curve (AUC) in Units (U) derived from the older AU * min (1U = 10 AU * min). Sample preparation and pipetting were done under standard laboratory conditions in laboratories in the Faculty of Natural Sciences, Comenius University, Bratislava. Measurement analysed by Multiplate^®^ Analyzer is dependent on the hematocrit level and platelet count such that extreme values of these parameters may result in an imprecise assessment of platelet function. However, no extreme values in both hematocrit levels and platelet count were detected in any sample.

### Statistical methods

Continuous variables are presented as sample means and standard deviations. The normality of data was assessed using a Shapiro–Wilk test and visually inspected on Q–Q plots. Repeated measures ANOVA was used to analyse concentration differences for each group and agonists separately. Student’s *t* test was used to compare differences in platelet aggregation response induced by ADP, ASPI, and TRAP with saline vs melatonin (10–5 M). Mixed linear model regression was used to analyse the effect of covariates (study group, age, sex and melatonin concentration) as well as the interaction of group (diabetic and control) and melatonin concentration on the platelet aggregation levels for each agonist separately.

Data were analysed using Python version 3.7.12 (https://www.python.org/) with appropriate libraries (for statistical analyses *pingouin* package version 0.5.0: https://pingouin-stats.org/).

### Sample size calculation

Platelet aggregation in the healthy population measured by Multiplate analyser was 68.6 ± 20.12 for ADP, 72.3 ± 18.08 for ASPI and 104.6 ± 19.60 for TRAP, respectively [27]. Expected reduction in platelet aggregation in response to melatonin is 30% in healthy individuals and 0% in diabetic patients [18]. With a minimal relevant difference of 20 U, a level of significance of 5% (alpha) and a power of 90%, (1-beta) we needed 13 sample pairs for ADP. With a minimal relevant difference of 22 U, a level of significance of 5% (alpha) and a power of 90%, (1-beta) we needed 10 sample pairs for ASPI. And with a minimal relevant difference of 31 U, a level of significance of 5% (alpha) and a power of 90%, (1-beta) we needed seven sample pairs for TRAP.

## Results

Our study was composed of two groups. The healthy control group (*n* = 15) included 11 males and 4 females with a mean age of 31.67 (ranging from 19 to 44, SD ± 7.28). Patients in this group had no relevant medical history.

Diabetic group (*n* = 15) included 4 males and 11 females with a mean age of 72.47 (ranging from 59 to 89, SD ± 10.12). Patients in this group had no relevant medical history. There was a statistically significant difference in age (*p* < 0.001) and sex (*p* < 0.001) between the groups (Table 1).

**Table 1:** Baseline data of diabetic and control group

	Diabetic	Control	*p* value
Age	72.47 ± 10.12	31.67 ± 7.28	< 0.001
Female sex	73.33%	26.67%	< 0.001

Since the data for all markers were normally distributed (ADP: *W* = 0.98, *p* = 0.25; ASPI: *W* = 0.99, *p* = 0.88; TRAP: *W* = 0.99, *p* = 0.67), we decided to use parametric statistical tests for subsequent analyses.

**Fig. 1:**
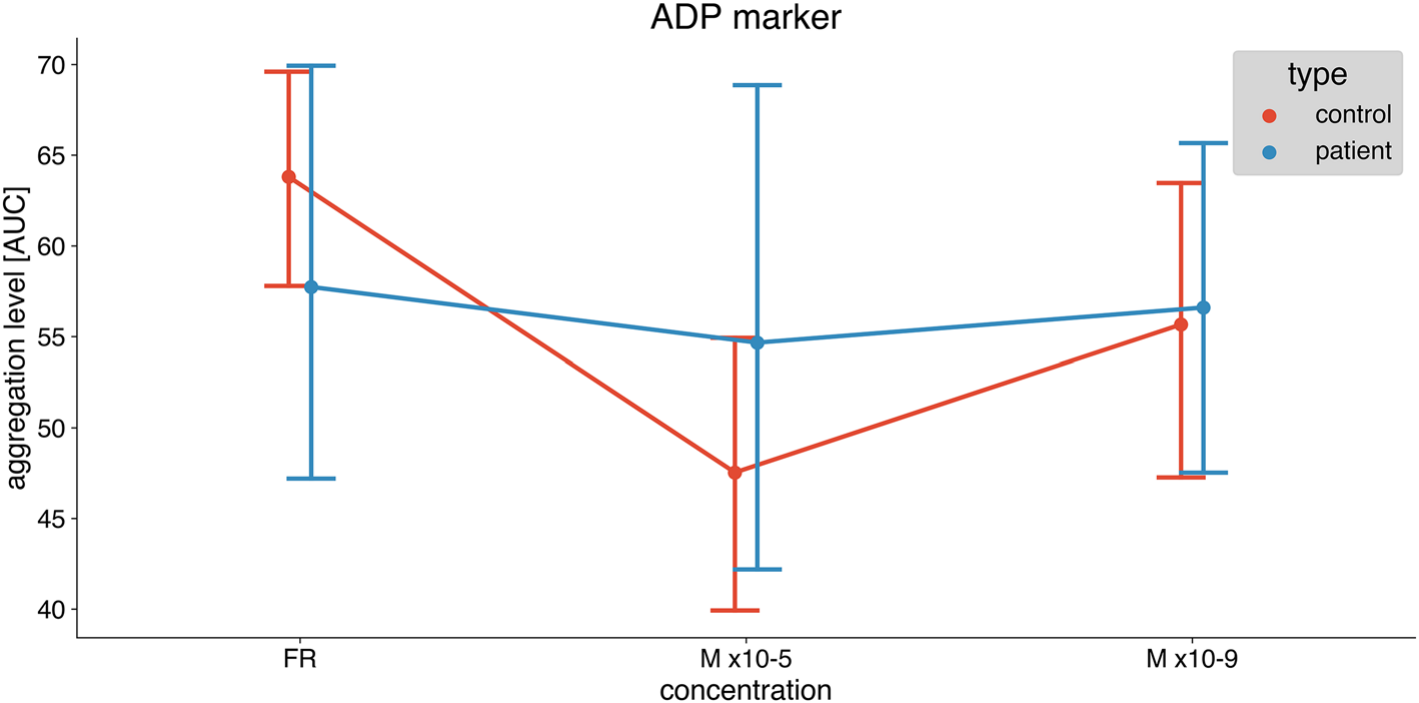
The difference in platelet aggregation response between healthy individuals (control) and diabetic patients induced by ADP: saline, melatonin 10–5 and melatonin 10–9 concentrations. Data are presented as mean with 95% confidence interval. Control group—*n* = 15, diabetic group—*n* = 15. *ADP* adenosine diphosphate, *AUC* area under the curve

**Fig. 2:**
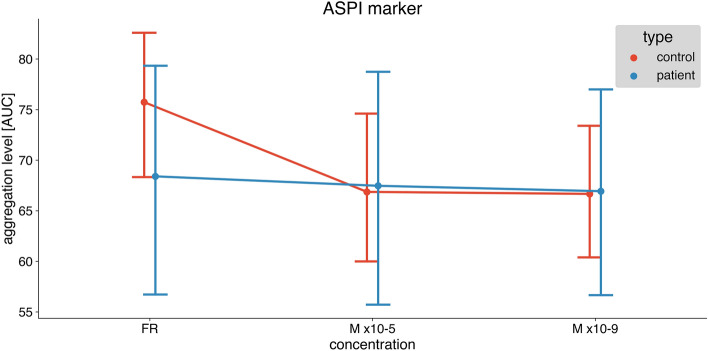
The difference in platelet aggregation response between healthy individuals (control) and diabetic patients induced by ASPI: saline, melatonin 10–5 and melatonin 10–9 concentrations. Data are presented as mean with a 95% confidence interval. Control group—*n* = 15, diabetic group—*n* = 15. *ASPI* arachidonic acid, *AUC* area under the curve

**Fig. 3:**
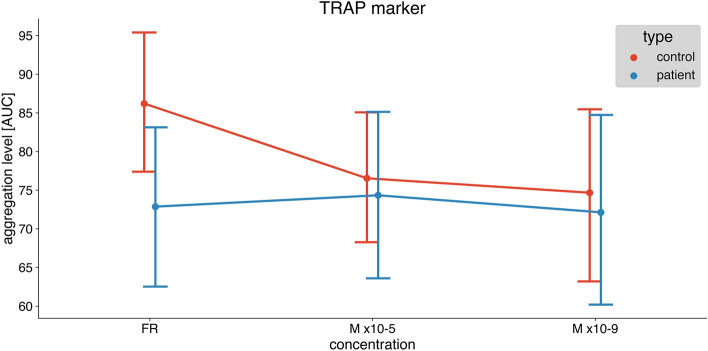
The difference in platelet aggregation response between healthy individuals (control) and diabetic patients induced by TRAP: saline, melatonin 10–5 and melatonin 10–9 concentrations. Data are presented as mean with a 95% confidence interval. Control group*—n* = 15*,* diabetic group—*n* = 15*.* TRAP thrombin, *AUC* area under the curve

In healthy individuals, melatonin significantly inhibited platelet aggregation both in higher (10–5 M) and lower concentrations (10–9 M) induced by ADP (Fig. 1), ASPI (Fig. 2), and TRAP (Fig. 3). Repeated measures ANOVA demonstrated statistically significant reductions (ADP: *p* < 0.001, ASPI: *p* = 0.002, TRAP: 0.029).

In samples from patients with DM melatonin did not affect platelet aggregation both in higher (10–5 M) and lower concentrations (10–9 M) induced by ADP (Fig. 1), ASPI (Fig. 2), and TRAP (Fig. 3). Repeated measures ANOVA demonstrated no statistically significant reduction (ADP: *p* = 0.579, ASPI: *p* = 0.871, TRAP: *p* = 0.757).

**Fig. 4:**
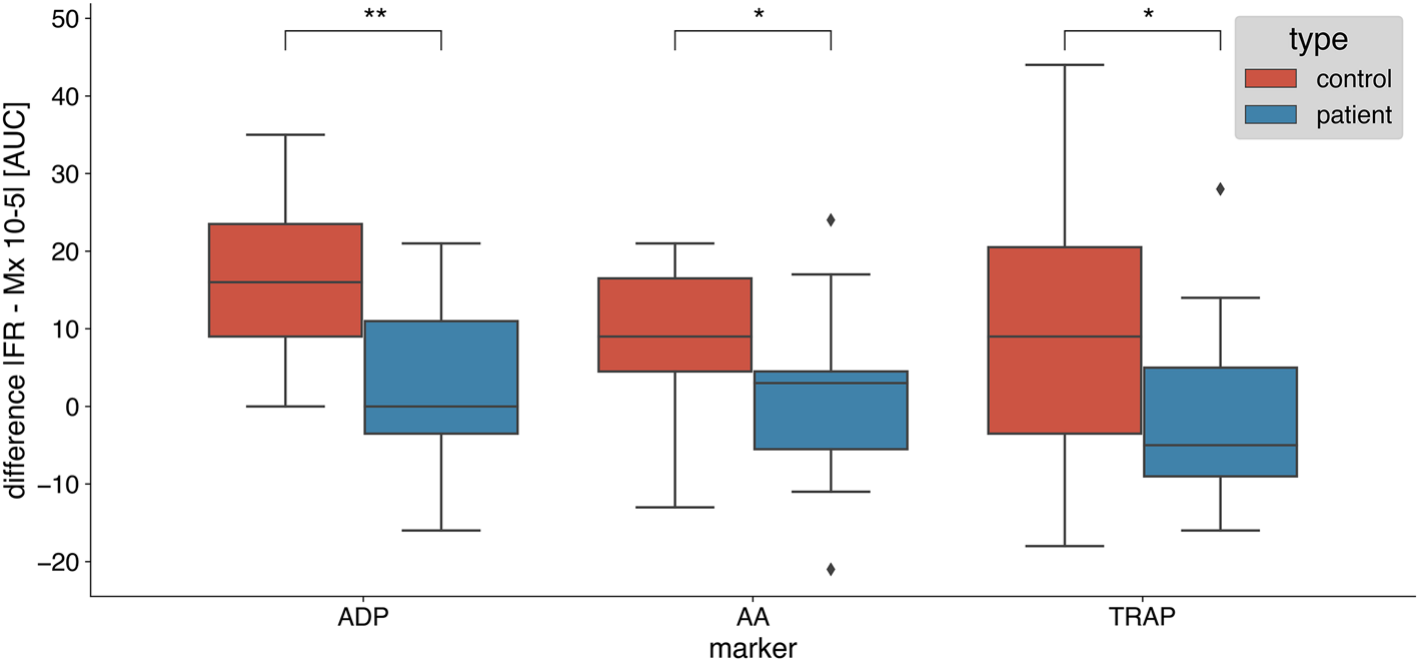
The difference in platelet aggregation response induced by ADP, ASPI, and TRAP with saline vs. melatonin (10–5 M). Control group*—n* = 15*,* diabetic group—*n* = 15*.* * = *p* value < 0.05, ** = *p* value < 0.01. *ADP* adenosine diphosphate, *ASPI* arachidonic acid, *TRAP* thrombin, *AUC* area under the curve

The difference in platelet aggregation response induced by ADP, ASPI, and TRAP with saline vs melatonin (10–5 M) was significantly higher in healthy individuals compared to patients with DM (*p* = 0.005, *p* = 0.045 and *p* = 0.048, respectively) (Fig. 4).

Mixed linear model regression models for TRAP (Table 2), ASPI (Table 3) and ADP (Table 4) showed a statistically significant effect of the study group (control vs. diabetic, *p* < 0.001, *p* = 0.014 and 0.006, respectively), 10–5 melatonin concentration (*p* = 0.01, *p* = 0.001 and *p* < 0.001, respectively), 10–9 melatonin concentration (*p* = 0.002, *p* = 0.001 and *p* = 0.007, respectively) and age (*p* < 0.001, *p* = 0.016 and *p* = 0.009, respectively) on platelet aggregation response. There was a statistically significant interaction between study group and 10–5 melatonin concentration (*p* = 0.035, *p* = 0.037 and *p* = 0.02) on platelet aggregation response. There was a statistically significant interaction between the study group and 10–9 melatonin concentration on platelet aggregation response for TRAP and ASPI (*p* = 0.041 and *p* = 0.037, respectively) but not for ADP (*p* = 0.098). There was no statistically significant effect of sex on platelet aggregation response induced by TRAP, ADP and ASPI (*p* = 0.618, *p* = 0.857 and 0.491, respectively).

**Table 2:** Mixed linear model regression model for TRAP

	Coef.	Std. Err.	*z*	*P* >|*z*|	[0.025	0.975]
Intercept	39.798	12.993	3.063	0.002	14.332	65.264
Diabetes	− 75.67	18.254	− 4.145	0	− 111.446	− 39.893
Melatonin concentration 10–5	− 9.667	3.735	− 2.588	0.01	− 16.987	− 2.346
Melatonin concentration 10–9	− 11.533	3.735	− 3.088	0.002	− 18.854	− 4.213
Sex	3.377	6.766	0.499	0.618	− 9.884	16.638
Diabetes and melatonin concentration 10–5 interaction	11.133	5.282	2.108	0.035	0.78	21.486
Diabetes and melatonin concentration 10–9 interaction	10.8	5.282	2.045	0.041	0.447	21.153
Age	1.3	0.349	3.728	0	0.616	1.983
Subject random effect	0.514	0.685	0.75	0.453	− 0.829	1.856

*Coef.* coefficient, *Std. Err.* standard error

**Table 3:** Mixed linear model regression model for ASPI

	Coef.	Std. Err.	*z*	*P* >|*z*|	[0.025	0.975]
Intercept	45.994	13.551	3.394	0.001	19.434	72.553
Diabetes	− 46.542	19.035	− 2.445	0.014	− 83.85	− 9.233
Melatonin concentration 10–5	− 8.867	2.685	− 3.302	0.001	− 14.13	− 3.604
Melatonin concentration 10–9	− 9.067	2.685	− 3.376	0.001	− 14.33	− 3.804
Sex	1.279	7.109	0.18	0.857	− 12.654	15.211
Diabetes and melatonin concentration 10–5 interaction	7.933	3.798	2.089	0.037	0.49	15.376
Diabetes and melatonin concentration 10–9 interaction	7.6	3.798	2.001	0.045	0.157	15.043
Age	0.881	0.366	2.406	0.016	0.163	1.599
Subject random effect	0.176	0.72	0.245	0.806	− 1.234	1.587

*Coef.* coefficient, *Std. Err.* standard error

**Table 4:** Mixed linear model regression models for ADP

	Coef.	Std. Err.	*z*	*P* >|*z*|	[0.025	0.975]
Intercept	29.849	13.46	2.218	0.027	3.468	56.231
Diabetes	− 51.757	18.908	− 2.737	0.006	− 88.816	− 14.697
Melatonin concentration 10–5	− 16.267	2.995	− 5.432	0	− 22.136	− 10.397
Melatonin concentration 10–9	− 8.133	2.995	− 2.716	0.007	− 14.003	− 2.264
Sex	4.851	7.049	0.688	0.491	− 8.964	18.667
Diabetes and melatonin concentration 10–5 interaction	13.2	4.235	3.117	0.002	4.899	21.501
Diabetes and melatonin concentration 10–9 interaction	7	4.235	1.653	0.098	− 1.301	15.301
Age	0.944	0.363	2.601	0.009	0.233	1.656
Subject random effect	0.325	0.714	0.455	0.649	− 1.074	1.723

*Coef*. coefficient, *Std. Err.* standard error

## Discussion

Our in vitro study demonstrated that melatonin significantly attenuates platelet aggregation induced by arachidonic acid, adenosine diphosphate and thrombin-receptor-activating-peptide in healthy patients’ whole blood. Blood was collected during the daytime when melatonin concentrations are very low, (below 1 pg mL^−1^). These concentrations are negligible compared to night-time levels, which we mimicked in our study. Moreover, published studies show that daytime melatonin levels do not significantly differ between nondiabetic individuals and diabetic patients [26]. This was true for higher (10–5 M) and lower (10–9 M) concentrations which are similar to the physiological concentrations in human blood [28]. In the whole blood of diabetic patients, melatonin was not associated with statistically significant differences in platelet aggregation. Finally, the attenuation of whole blood platelet aggregation induced by melatonin was significantly higher in healthy people compared to diabetic patients.

In the bivariate analysis, there was a statistically significant difference in both age and sex between the two study groups. Mixed linear model regression was used to analyse the effect of these covariates on platelet aggregation induced by ADP, ASPI and TRAP. This analysis confirmed attenuated response to melatonin in diabetic patients in all experiments except for lower (10–9 M) concentration for ADP. Sex was not a significant contributor contrary to previously reported data. A review study by Carazo et al. shows that out of 78 reviewed papers 68 reports a sex-related difference in platelet aggregation [29]. Higher platelet reactivity in women is probably affected via multiple COX‐1–dependent and COX‐1–independent pathways [30]. On the other hand, age was a significant contributor to platelet aggregation also in this multivariate analysis. The effect of aging on platelet aggregation is complex and probably influenced by many factors including oxidative stress, age-related plasma membrane modifications, alterations in platelet-serotonin system, vascular prostaglandin secretion, transcriptome, hormonal changes and the effect of coexisting diseases [31].

Previous studies reported that melatonin inhibits platelet aggregation that is induced by ADP or ASPI [19, 20], and we demonstrated that melatonin also inhibits aggregation induced by thrombin. However, there are no melatonin receptors in thrombocytes [32], and the antiplatelet mechanism of melatonin is unknown. The three most important activators of platelet aggregation are ASPI, ADP and thrombin which were used in our study as prothrombotic inductors. Because melatonin in healthy people attenuates aggregation in all three of these activators, there is little likelihood that melatonin exerts its antiplatelet effects via one of these pathways. Besides known effects of melatonin on platelet aggregation a study on animal models by Hajam et al. demonstrated also that melatonin treatment restores impairments in the antioxidative system, serum electrolytes, cellular total protein, glycogen content and histoarchitecture of liver and kidney cortex caused by diabetes. The novelty of our study lies in the previously undescribed altered effect of melatonin in diabetic patients [33].

Except for triggering melatonin receptors, melatonin also activates proliferator-activated receptor (PPAR) α and γ [34–37]. It is known that PPAR stimulation causes increased intraplatelet cAMP, negative regulation of αIIbβ3 integrinand subsequent inhibition of platelet aggregation [38, 39].

One study reports that melatonin suppresses platelet aggregation via activation and restoration of PPARγ in platelets, which play an important role in FUNDC1‐required mitophagy, mitochondrial energy production, platelet hyperactivity, and cardiac I/R injury [40]. This might explain the antithrombotic effects of melatonin in healthy participants.

In our in vitro study melatonin did not affect platelet aggregation in patients with DM. The night-time antiplatelet mechanism of melatonin is missing in DM patients and might be responsible for their absence of AMI circadian variation.

One explanation for this phenomenon is the alteration of the PPAR signalling pathway seen in diabetes and hyperglycaemia. The transcriptional network mediated by FoxO1/PPARγ functions as a key element in pancreatic β-cell adaptation to metabolic stress with important regulatory control over glucose and mitochondrial metabolism, prodifferentiation, incretin effects, and β-cell compensation to obesity and insulin resistance. Failure of this response is responsible for the onset or exacerbation of diabetes. Furthermore, excessive expression of pro-inflammatory cytokines suppresses PPARγ activity causing abnormalities of the wnt/β-catenin, lysosomal acid lipase, plasminogen activator system, inflammatory and cell cycle pathways [41, 42].

This hypothesised relationship is further supported by the results from clinical trials of PPAR agonists. For example, PPARγ activation by pioglitazone reduced the incidence of AMI or stroke in patients with insulin resistance however, according to the prespecified sub-analysis the beneficial effect was present especially in patients with lesser grade insulin resistance (HOMA-IR < 4.6) and lower glycated haemoglobin concentrations (HBA_1C_ < 5.7%) [43]. On the other hand, when rosiglitazone was given to patients with DM, a significant increase in AMI risk observed [44]. A similar situation was observed with fibrates which are PPARα agonists. In patients without DM, gemfibrozil showed significant reductions in MI and stroke [45, 46]. However, in patients with DM fenofibrate had no effect on these thrombotic events[47, 48].

This is the first study to demonstrate in vitro that the antiplatelet effect of melatonin in patients with type 2 DM is significantly attenuated, possibly explaining their absence of circadian variation in AMI incidence. Whether this finding contributes to the etiopathogenesis of the prothrombotic state in patients with DM merits further research. Understanding the exact mechanism of platelet resistance to melatonin in diabetic patients would permit a better understanding of the disease pathophysiology and also the consideration of new therapeutic and diagnostic options such as therapeutically targeting the dysfunctional signalling pathways in diabetic patients or testing the degree of resistance to melatonin to stratify patient risk. A precise understanding of PPAR pathway and the influence of individual signalling molecules would allow using PPAR agonists to reduce the risk of MI and stroke in precisely defined patient groups.

## Study limitations

This study was performed as an in vitro experiment and although physiological concentration (M-9) of melatonin was used the results cannot be directly extrapolated to in vivo pathophysiology. Patients were not matched in the study groups therefore, other factors besides the presence of type 2 diabetes mellitus and age such as menstrual cycle, hormonal contraception, or differences in body mass index might have affected the platelet aggregability.

## Data Availability

Raw data supporting the conclusions of this article are available from the corresponding author upon reasonable request.
